# Neuroprotection with hypothermia and allopurinol in an animal model of hypoxic-ischemic injury: Is it a gender question?

**DOI:** 10.1371/journal.pone.0184643

**Published:** 2017-09-20

**Authors:** Javier Rodríguez-Fanjul, Cristina Durán Fernández-Feijóo, Míriam Lopez-Abad, Maria Goretti Lopez Ramos, Rafael Balada Caballé, Soledad Alcántara-Horillo, Marta Camprubí Camprubí

**Affiliations:** 1 Department of Neonatology, BCNatal, Sant Joan de Déu-Hospital Clínic, Barcelona, Spain; 2 Department of Neonatology, Hospital Álvaro Cunqueiro, EOXI, Vigo, Spain; 3 Department of Pathology and Experimental Therapeutics, Faculty of Medicine and Biomedical Sciences, Bellvitge Campus, University of Barcelona, Barcelona, Spain; Hopital Robert Debre, FRANCE

## Abstract

**Background:**

Hypoxic-ischemic encephalopathy (HIE) is one of the most important causes of neonatal brain injury. Therapeutic hypothermia (TH) is the standard treatment for term newborns after perinatal hypoxic ischemic injury (HI). Despite this, TH does not provide complete neuroprotection. Allopurinol seems to be a good neuroprotector in several animal studies, but it has never been tested in combination with hypothermia.

Clinical findings show that male infants with (HI) fare more poorly than matched females in cognitive outcomes. However, there are few studies about neuroprotection taking gender into account in the results.

The aim of the present study was to evaluate the potential additive neuroprotective effect of allopurinol when administrated in association with TH in a rodent model of moderate HI. Gender differences in neuroprotection were also evaluated.

**Methods:**

P10 male and female rat pups were subjected to HI (Vannucci model) and randomized into five groups: sham intervention (Control), no treatment (HI), hypothermia (HIH), allopurinol (HIA), and dual therapy (hypothermia and allopurinol) (HIHA). To evaluate a treatment’s neuroprotective efficiency, 24 hours after the HI event caspase3 activation was measured. Damaged area and hippocampal volume were also measured 72 hours after the HI event. Negative geotaxis test was performed to evaluate early neurobehavioral reflexes. Learning and spatial memory were assessed via Morris Water Maze (MWM) test at 25 days of life.

**Results:**

Damaged area and hippocampal volume were different among treatment groups (p = 0.001). The largest tissue lesion was observed in the HI group, followed by HIA. There were no differences between control, HIH, and HIHA. When learning process was analyzed, no differences were found. Females from the HIA group had similar results to the HIH and HIHA groups.

Cleaved caspase 3 expression was increased in both HI and HIA. Despite this, in females cleaved caspase-3 was only differently increased in the HI group.

All treated animals present an improvement in short-term (Negative geotaxis) and long-term (WMT) functional tests. Despite this, treated females present better long-term outcome. In short-term outcome no sex differences were observed.

**Conclusions:**

Our results suggest that dual therapy confers great neuroprotection after an HI event. There were functional, histological, and molecular improvements in all treated groups. These differences were more important in females than in males. No statistically significant differences were found between HIHA and HIH; both of them present a great improvement. Our results support the idea of different regulation mechanisms and pathways of cell death, depending on gender.

## Introduction

Hypoxic-ischemic encephalopathy (HIE) is one of the most important causes of neonatal brain injury. It is associated with high mortality and long-term neurological sequelae [1]. Its global incidence is around 1–3 per 1000 term births in developed countries [2] with a mortality rate between 10–15% in those infants with moderate to severe disease. Among the survivors, at least 36% will present severe neurodevelopmental disabilities and a larger proportion (30%) will present significant problems including global developmental delay, cognitive problems, deafness, and epilepsy [3,4].

Therapeutic hypothermia (TH) has been the only proven treatment for term newborns after perinatal HIE injury [4]. The mechanisms underlying hypothermia neuroprotection are multifactorial. Suppression of excitotoxicity [5], decreasing oxidative stress [6], and inflammation [7] modulation of intracellular signaling and programmed cell death [8] seem to be some of the most important. Although several randomized controlled trials have shown that TH reduces mortality and improves neurodevelopmental outcome among survivors [9,10,11], this therapy does not provide complete neuroprotection [12]. Therefore, new therapeutic approaches to reduce brain injury are necessary. After an HI event, there is a considerable increase in free radical production, lending support to the idea that oxidative stress plays a key role in the pathology of secondary brain injury [13]. Taking all the foregoing into account, it seems reasonable to try to reduce this secondary damage using antioxidant therapies [14,15] in combination with hypothermia [16].

Some of the most important sources of oxygen free radicals are xanthine oxidase products.

Allopurinol is a xanthine oxidase inhibitor, one of the main prooxidant pathways after HI that inhibits the conversion of hypoxanthine into xanthine and uric acid, thereby limiting the toxic overproduction of ROS. It prevents adenosine degradation and oxygen radical formation, and preserves NMDA receptor integrity, so as a consequence it may reduce brain injury in HIE through several mechanisms of action which are independent of the proven beneficial effect of hypothermic treatment in cellular energy metabolism. In high concentrations, it is also a direct scavenger of the toxic hydroxyl free radical and a chelator of non-protein-bound iron [17].

The first reports of the neuroprotective effects of allopurinol in animal models were published in the early 1990s [18,19]. Initial histology and molecular data showed promising results [20,21], and when allopurinol was tested in humans some beneficial effects were demonstrated on free radical formation, cerebral perfusion, and electrical brain activity, without toxic side effects [22,23]. Despite this, Benders and colleagues, who only included severely asphyxiated infants, concluded that no beneficial effects were detected in the short-term clinical evaluation regardless of its higher safety profile [24,25,26,27]. However, when the long-term neurological outcome of these patients was evaluated, those with moderate HIE treated with allopurinol presented better cognition results, suggesting that neonatal allopurinol treatment may improve long-term results in moderate HIE newborns [25].

Along the same line, a randomized controlled trial focusing on prenatal administration of the drug (NCT001189007) demonstrated that maternal treatment with allopurinol during fetal hypoxia did not significantly lower neuronal damage markers in cord blood. Nonetheless, the *post hoc* analysis revealed a potential beneficial effect in girls [28].

There is increasing evidence of sex differences in HI outcomes, with males exhibiting more severe neurological deficits relative to matched females [29]. *In vitro*, it has been demonstrated that mechanisms of cell death and brain lesion have different sex injury pathways [30, 31]. Taking all this into account, it is reasonable to make examine sex-specific treatment effects.

The aim of the present study was to evaluate the effects of combining hypothermia and allopurinol in an animal model of HI brain injury. Sex differences in neuroprotection were also evaluated.

## Material and methods

### Procedures

This study was carried out in strict accordance with the recommendations in the Guide for the Care and Use of Laboratory Animals of the National Institutes of Health. The protocol was approved by the Ethics Committee for Animal Experimentation of the University of Barcelona (Permit Number: 6575), following European (2010/63/UE) and Spanish (RD 53/2013) regulations for the care and use of laboratory animals. All surgery was performed under inhaled isofluorane, and all efforts were made to minimize the animals’ suffering and the number of them used to carry out the experiments.

### Animals and ethics statement

Postnatal day 10 (P10) Wistar rats (HARLAM, Netherlands) were used in this study. After birth, animals were kept with their mothers in cages with 12-hour light/dark cycles at a constant temperature of 22 ± 1°C with free access to food and water. All surgical and experimental procedures were in accordance with the international recommendations of the Guide for the Care and Use of Laboratory animals. Experimental procedures were approved by the local ethical committee of the University of Barcelona (Permit Number: 6575), following European (2010/63/UE) and Spanish (RD 53/2013) regulations for the care and use of laboratory animals. All surgery was performed under inhaled isofluorane, and all efforts were made to minimize the animals’ suffering and the number of them used.

Animals were euthanized prior to the end of the experiments by the administration of intraperitoneal thiopental after being anesthetized with inhaled isofluorane (2%).

#### Hypoxia-ischemia animal model

P10 pups were randomized into 5 experimental groups. Neonatal Hypoxia Ischemia (HI) was induced using the Rice-Vannucci model. Briefly, unilateral ligation of the left common carotid artery was performed and afterwards animals were exposed to 90 minutes of hypoxia (8% oxygen atmosphere), as previously described [26].

At the end of the HI, pups were treated with systemic hypothermia (32.5–33°C) or normothermia (36–36.5°C) in temperature-controlled chambers for 5 hours. Temperature was continuosly measured in one pup in each chamber with a rectal temperature probe (IT-21; Physitemp Instruments).

Sham-operated animals were anesthethized and a skin incision was performed to expose the left common carotid artery but without artery ligation or hypoxia.

All treatment groups received a single intraperitoneal injection of allopurinol (Zylosprim sodium, Burroughs Wellcome, Research Triangle Park, NC) at 135 mg/kg (volume: 0.01 ml/g) or saline, 15 min after hypoxia, depending on the randomization, before beginning hypothermia or normothermia protocol. Allopurinol doses were the same as that used by Palmer et al [19].

#### Animal randomization and experimental design

Five experimental groups were established for global evaluation.

Animals were randomized in: *Sham-treated* (control), *HI+normothermia* (HI), *HI+allopurinol* (HIA), *HI+hypothermia* (HIH), and *HI+hypothermia+ allopurinol* (HIHA).

**Fig 1:**
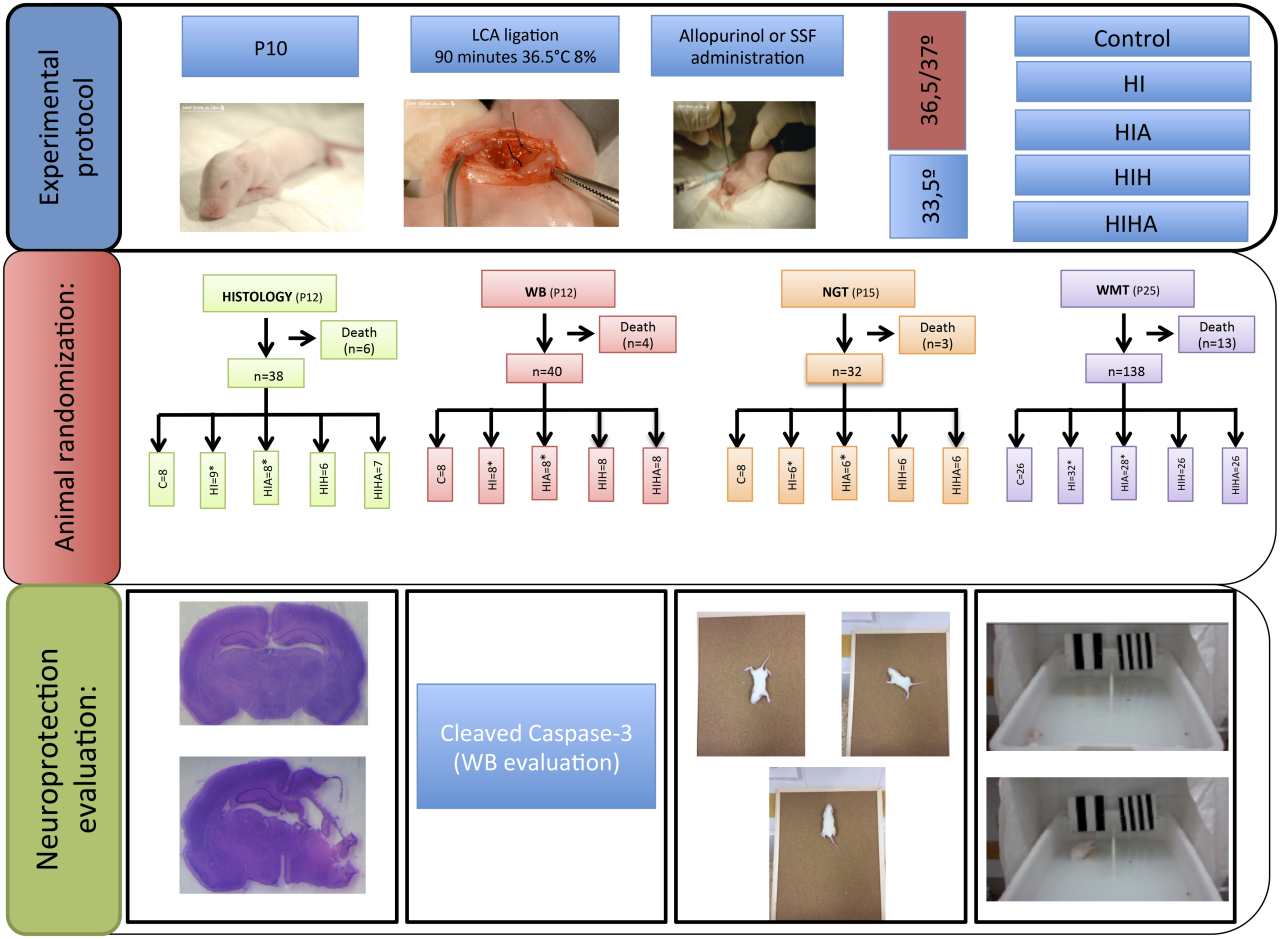
Study diagram. P10: 10 days of life, LCA: Left common carotid, ST: Sham-treated; HI: Hypoxic-ischemic, HIA: Hypoxic-ischemic allopurinol, HIH: Hypoxic-ischemic hypothermia, HIHA: Hypoxic-ischemic hypothermia allopurinol, HE: Histological evaluation, MWMT: Water maze test.

A diagram of the experimental design including the number of animals used for each condition and analysis is presented in Fig 1.

### Histological evaluation: Measurement of infarct and hippocampal volume

Pups were deeply anesthetized with isofluorane and perfused transcardically with 4% paraformaldehyde 72 hours after the HI injury. Brains were carefully removed and maintained for 12 hours in the same solution at 4°C, and then cryoprotected in 30% Sucrose/PBS at 4°C for 24-48h, and stored at -80°C. Forty μm coronal sections were made and stained with hematoxylin-eosin. The midline of each brain section was identified on the image and the brain divided by hemispheres (left vs right). Two sections from each block representing cortex, hippocampus, basal ganglia, and thalamus were scanned at 1200 dpi resolution. ImageJ (ImageJ, National Institutes of Health, USA; www.NIH.gov) was used to measure the area of viable tissue in the left and right hemispheres. The ratio of the measured brain area was calculated for the two sections per brain and the average percentage of area loss was calculated (1–(Area Ratio (right vs left)) [16]

Hippocampal volume was analysed comparing the affected hippocampal side with the contralateral hippocampus.

#### Neuropathological score

Thorensen neuropathological score was used for the cerebral cortex and hippocampus. The evaluation was performed at the same time as the histologial evaluation. In the cerebral cortex, scoring was performed as follows: *0*: no histological damage; *1*: < 10% affected area; small, patchy or incomplete infarcts; *2*: 20–30% affected area; partly confluent infarcts; *3*: 40–60% affected area, with large confluent infarcts; *4*: > 75% affected area; total disaggregation of the tissue.

Findings were: in the hippocampus *1*: <20% affected area, necrotic neurons only in the most lateral areas; *2*: 50% affected area, patchy areas in all sectors; *3*: 75% affected area, with more extensive areas of necrotic neurons; *4*: 100% affected, complete infarction of hippocampus including gyrus dentatus [27].

### Western blot

Pups were sacrificed by decapitation 24 hours after the HI injury and their brains were rapidly removed and weighed. Protein extracts from the right hippocampus were separated by SDS-PAGE and electro-transferred into a nitrocellulose membrane. Membranes were blocked with 5% nonfat dry milk in Tris-buffered saline and incubated first with primary antibodies against cleaved caspase-3 ((Asp175) (5A1E) Rabbit #Cell signaling, 1/500) or GAPDH (D16H11) XP Rabbit #Cell signaling, 1/20000) overnight at 4°C, and then with their corresponding HRP-conjugated secondary antibody (PROMEGA anti-Rabbit 1/5000). Protein signal was detected using the ECL chemiluminescent system (Amersham, Buckinghamshire, UK).

### Negative geotaxis

On P15, 5 days after the HI insult, the animals underwent the ‘negative geotaxis’ test, which examines the time taken to rotate 180° from a head-down to a head-up position when placed head down on a 45° slope [32]. This is an innate postural response that appears in the second week of life in normal pups.

### Functional evaluation: Water maze test

Spatial memory evaluation was carried out in a version of Morris’s water maze pool (MWM) [33] modified for small animals (63 cm l, 43 cm wide, and 35 cm high), with the water temperature set at 22–23°C and water made opaque by latex suspension (Fig 1). The escape latency (EL), defined as the time taken to reach the platform, was measured during each trial as an indicator of learning. At P25, all pups from each experimental group were trained four times per day, for 10 consecutive days.

### Statistical analysis

Each litter was randomized to include pups of each sex and treatment group. For histological analysis, western blot and negative geotaxis test, a minimum of *n* = 4 per sex to observe a 50% difference by Wilcoxon signed rank test (80% power, *a* = 0.05) was required. This figure was based on previous studies with antioxidant treatments [34]. For WMT, at least 6 animals per sex and per condition were necessary, as reported in other studies.

The anthropometric results were expressed as mean and range. Infarct and hippocampal volume were expressed as mean ± SEM. Statistical differences were analyzed using one-way ANOVA followed by Turkey multiple comparison procedure. For western blot, the values were expressed as a ratio of GAPDH and then converted to percentage of control group (presented as bar diagrams) and represented as mean ± SEM. Due to problems with ascertainment of normal distribution in small sample sizes, data were also analyzed with non-parametric tests (Kruskal-Wallis test) which yielded identical results and statistical significance. Negative geotaxis test was analysed using a Kruskall-wallis test. The escape latency of rats in the MWM training was analysed using two-way ANOVA (time, group) with repeated measures, corrected with Bonferroni adjustments. A criterion of p <0.05 was considered significant. All analyses were performed using Stata 13.

## Results

### Survival rates, weights, and cephalization index

A total of 260 animals were used during the whole experiment. Twenty-two animals (9.7%) died after the procedure, before being randomized. After the treatment, there were 4 casualties in the HI and 2 in the HIA. None of the animals treated with HIH or HIHA died. There was a significant difference in weight evolution between treated animals and those in the HI group, which were smaller (P = 0.004).

### Histopathological analysis

**Fig 2:**
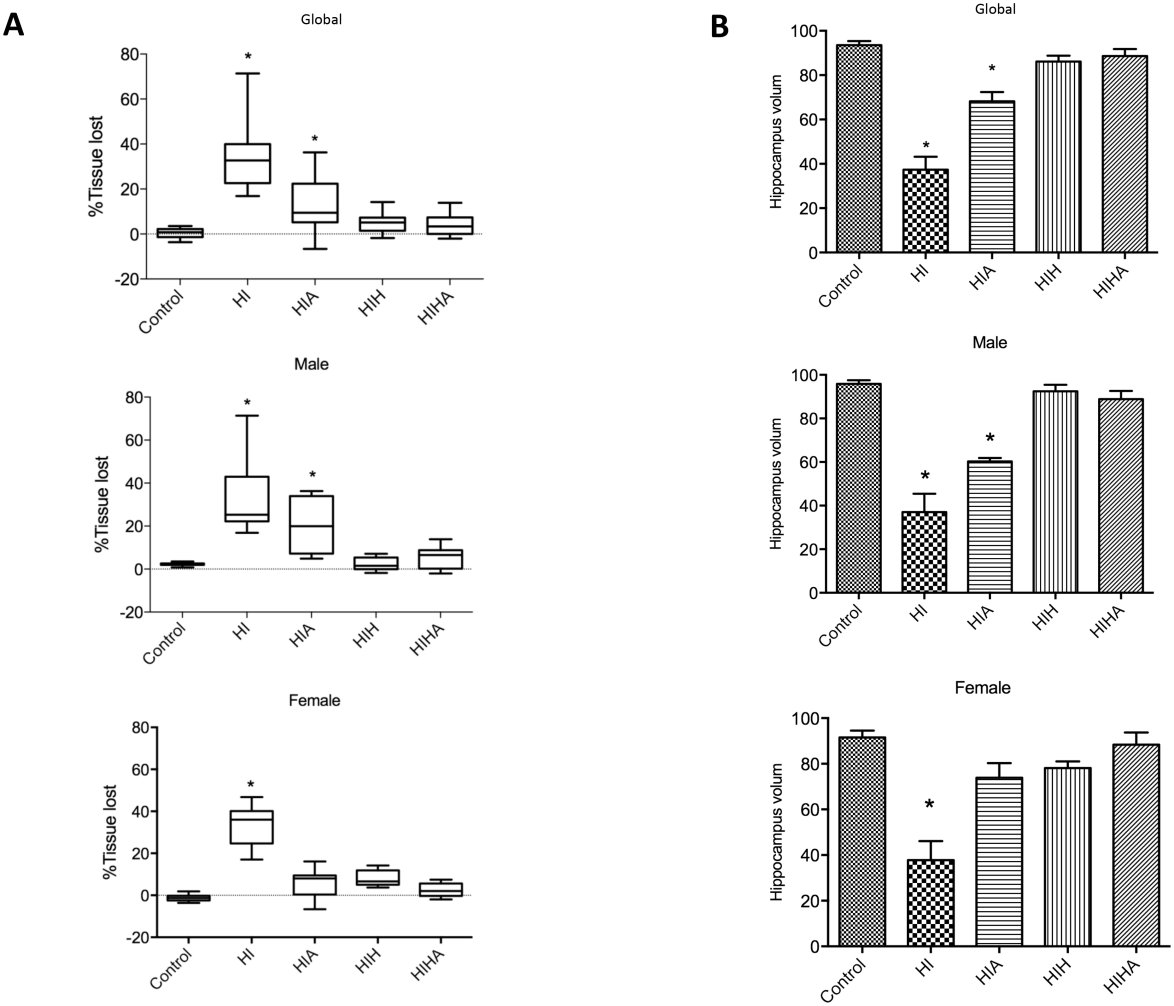
Histological evaluation. A: Graphical representation of the percentage of brain area lost in the affected hemisphere with respect to the contralateral hemisphere by experimental group (global), and by group and gender (Male, Female) B: Graphical representation of the hippocampal volume in the affected hemisphere by experimental group (global), and by group and gender (male, female) ST: Sham-treated; HI: Hypoxic-ischemic, HIA: Hypoxic-ischemic allopurinol, HIH: Hypoxic-ischemic hypothermia, HIHA: Hypoxic-ischemic hypothermia allopurinol. Volume areas are expressed in arbitrary units. * Significant differences p<0.05.

Histological evaluation was performed 72 hours after the HI event, in coronal sections stained with hematoxylin-eosin. The mean area loss in the untreated group was 33.60% (±3.30). The mean area losses in the different treatment groups were: HIA 13.41% (±3.37); HIH, 5.1% (±1.04); HIHA, 3.8% (±0.99). Mean area loss in the control group was 0.37% (±0.5). Infarct area was reduced in treated animals (HIA HIH, HIHA) with respect to the HI untreated group (p<0.0001) (Fig 2A). No statistically significant differences were found between HIH and HIHA (Fig 2A).

When whole infarct area was analysed by sex, there were no statistically significant differences between males and females (p = 0.321). But when it was analyzed by sex and condition, HIA females exhibited a smaller infarct volume than HI females (p = 0.037). Moreover, there were no differences in infarct volume among females from the HIA, HIH, and HIHA groups (p = 0.151; p = 1; p = 1) (Fig 2A). In males, infarct area was reduced in HIH and HIHA, but no stadistically significant differences were found between HIA and HI (Fig 2A).

There were also differences in hippocampal volume between treatment groups (p<0.0001). HI animals without treatment presented a smaller hippocampus when compared to the others while there were no differences between the HIH, HIHA, and control groups (p = 1) (Fig 2B).

When sex was considered, there were no differences in the hippocampal volume between HIA, HIH, and HIHA in females (p = 0.398; p = 1; p = 1). Meanwhile in males, the hippocampal volume of those animals treated with allopurinol was similar to the HI ones, and different from the others (p = 0.003) (Fig 2B).

### Neuropathological score

Neuropathological score was different in the groups (p = 0.001). In the post-hoc analysis, control (p = 0.001), HIH (p = 0.001), and HIHA (p = 0.001) were different from HI and HIA. Histological structure was better preserved in control and hypothermia-treated animals (HIH and HIHA) than in HI and HIA, in which histological damage was more evident, with large infarcts and affected areas.

**Fig 3:**
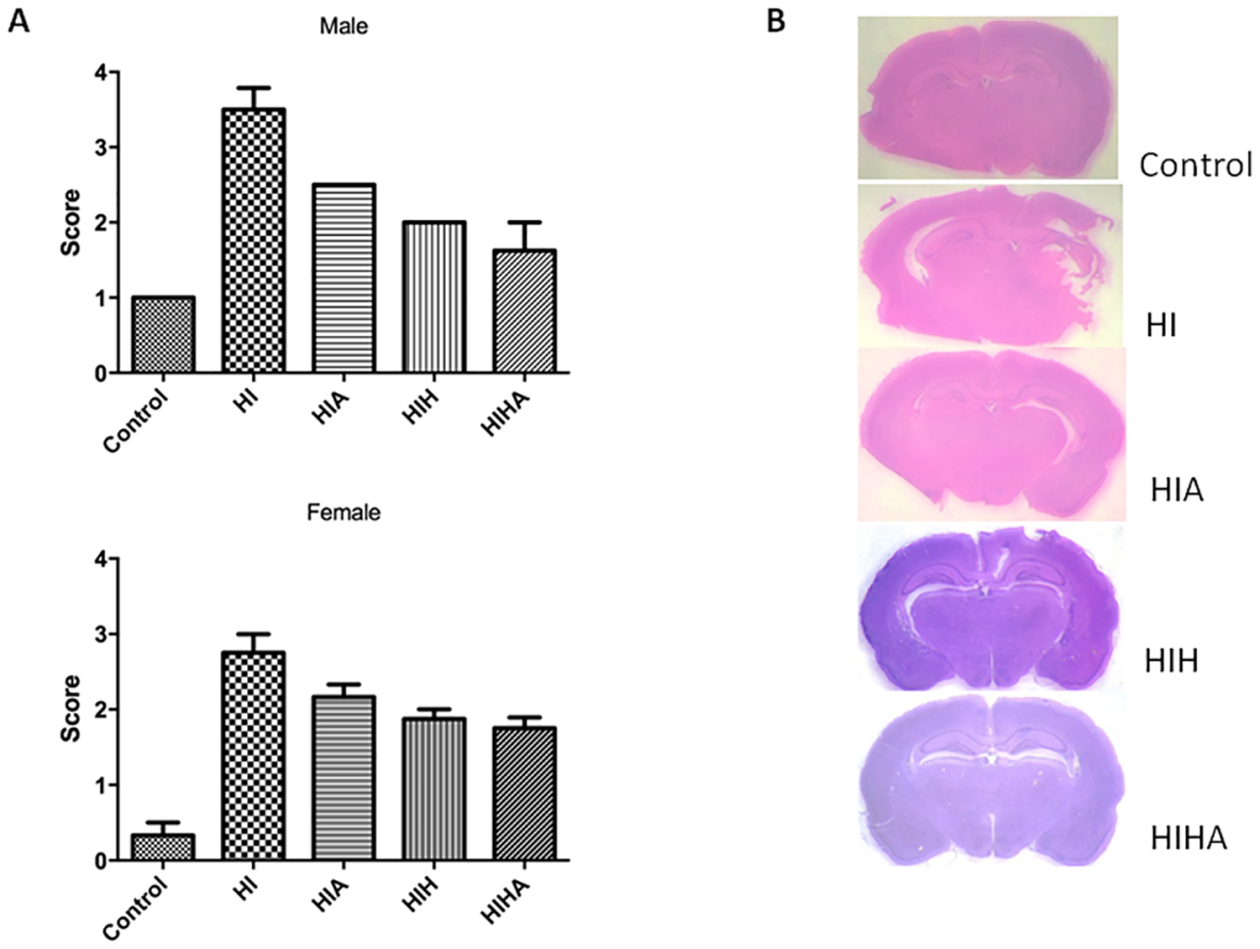
Macroscopic-microscopic histologic evaluation. A: Neuropathological brain scores. B: Representative photograph of perinatal coronal brain sections of the different experimental groups. ST: Sham-treated; HI: Hypoxic-ischemic, HIA: Hypoxic-ischemic allopurinol, HIH: Hypoxic-ischemic hypothermia, HIHA: Hypoxic-ischemic hypothermia allopurinol.

When gender was taken into account, there were also differences in males between groups (p = 0.0004); control (p = 0.001), HIH (p = 0.0004), and HIHA (p = 0.0006) were different from HI and HIA. In females, there were also differences among all the groups (p = 0.003), but control (p = 0.02) HIH (p = 0.036), HIHA (p = 0.036), and HIA (p = 0.029) were different from HI (Fig 3).

No differences were detected between HIH and HIHA groups (p = 0.8039).

### Cleaved caspase-3 activation

**Fig 4:**
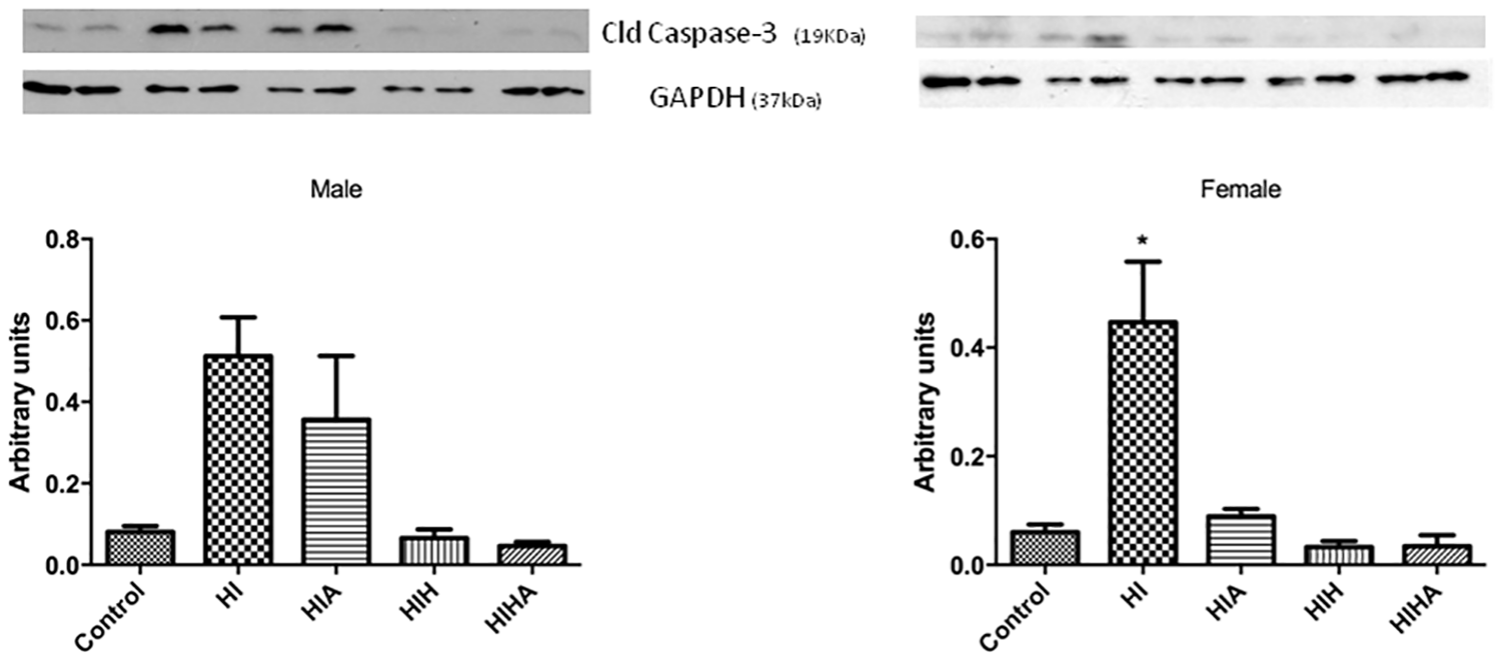
Cleaved caspase-3 expression. Western blot and densitometry analysis of cleaved caspase-3 protein in the hippocampal tissue of males and females from the different experimental groups. GAPDH was used as protein loading control. Abbreviatons: Control; HI: Hypoxic-ischemic, HIA: Hypoxic-ischemic allopurinol, HIH: Hypoxic-ischemic hypothermia, HIHA: Hypoxic-ischemic hypothermia allopurinol. * Significant differences p<0.05.

To determine whether the observed sex- and treatment-dependent reduction in the infarcted area was due to early protection against apoptosis, the level of cleaved caspase-3, an indicator of apoptosis, was evaluated with western blot (Fig 4). At 24h post-HI, males showed increased levels of cleaved caspase-3 in the HI and HIA groups compared to controls (p = 0.0032). Meanwhile cleaved caspase-3 levels remained at control levels in the other treatment groups (HIH and HIHA). In contrast, in females, cleaved caspase-3 was only differently increased in the HI group (p = 0.0117) while it remained at control levels in the HIA, HIH, and HIHA groups, which showed reduced cleaved caspase-3 levels when compared to the HI group (HIHA p = 0.0249, HIA p = 0.046, HIH p = 0.025). No differences were detected between the HIH and HIHA groups (p = 0.4982).

### Negative geotaxis test evaluation

Negative geotaxis test was different between groups (p = 0.002). All treatment groups presented better results than non-treated animals. When gender was taken into account these differences were also the same. (See supporting information)

### Water maze test evaluation

**Fig 5:**
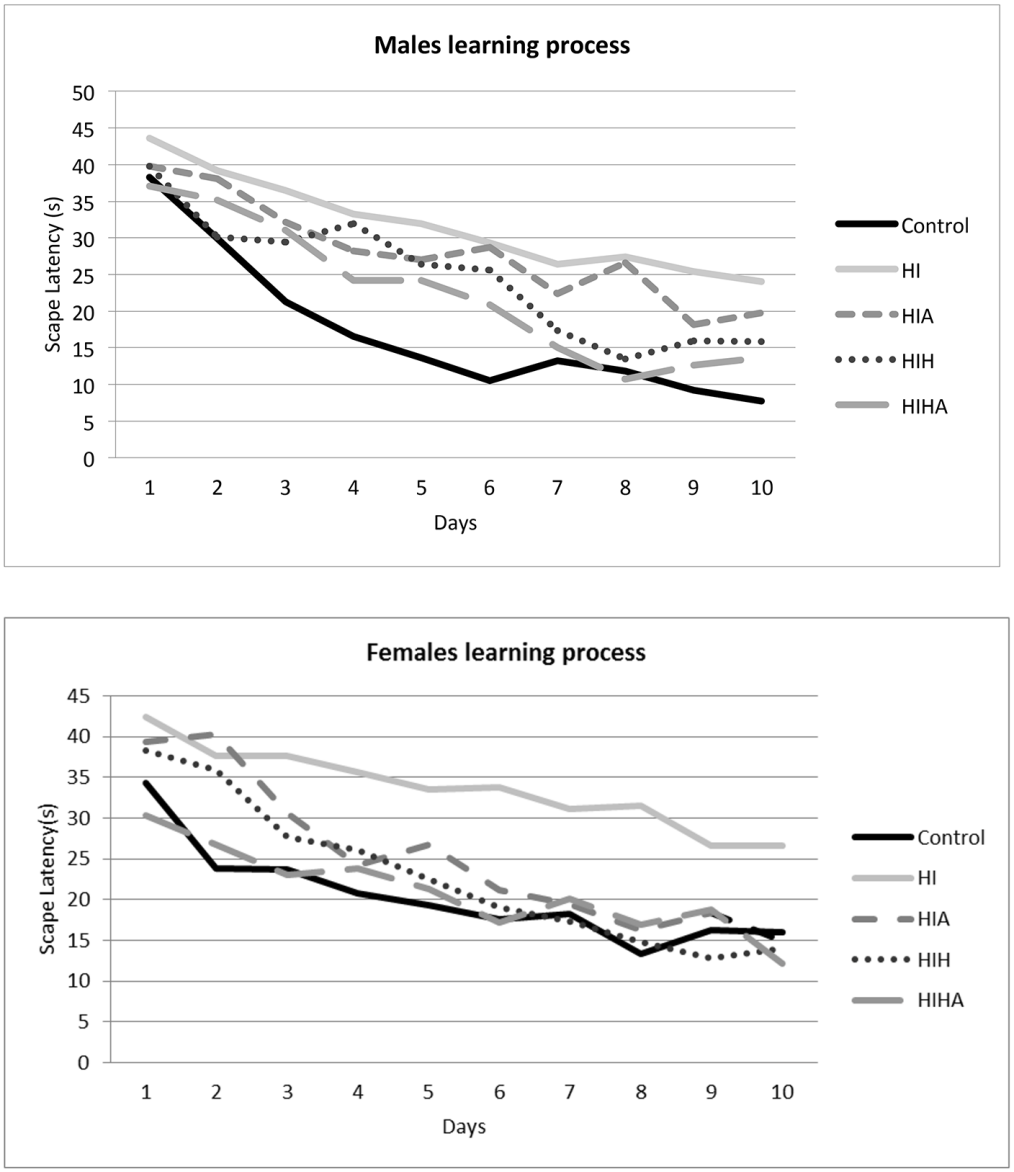
Water maze test. Plot representing the average escape latency in four trials performed each day. Results were expressed as mean of escape latency. Abbreviatons: ST: Sham-treated; HI: Hypoxic-ischemic, HIA: Hypoxic-ischemic allopurinol, HIH: Hypoxic-ischemic hypothermia, HIHA: Hypoxic-ischemic hypothermia allopurinol.

The Morris water maze test was used to evaluate the effects of the different treatments on learning and spatial memory. For each animal, escape latency (EL) was recorded 4 times a day for ten consecutive days, and the results were analyzed with two-way ANOVA. A graphical representation of learning performance by group and sex is shown in Fig 5. All groups acquired the task and improved their performance over time (p<0.0001), but with significant differences in the learning process, with the HI group being the worst.

*Post hoc* analysis revealed no differences in learning process among the control, HIH (p = 0.181), and HIHA (p = 0.709) groups, which were better than HI (p = 0.001) and HIA (p = 0.002).

Gender was also analysed as a possible confounding learning factor, but no differences were found in learning process between males and females (p = 0.965).

Learning progression was then analysed by gender and condition. In males, global learning was different between treatment groups (p = 0.001), but there were no differences between control, HIH (p = 0.18), and HIHA (p = 0.502). Meanwhile for HIA and HI the learning process was significantly worse than for the other groups (HIH, HIHA, and controls) (p = 0.0001) (p = 0.008).

In females, global learning was also different between groups (p = 0.001). When learning process was analysed by treatment only the HI group showed significantly poorer scores (p = 0.001), while control, HIH (p = 0.999), HIHA (p = 0.991), and HIA (p = 0.719) showed normal learning performance.

In summary, HIHA is a good neuroprotective strategy, although in males it does not add many benefits to HIH. Our results indicate that in females, HIA and HIHA increase neuroprotection, reducing infarct volume and preserving hippocampus. Moreover, their neuroprotective effect was long lasting, as learning outcomes were significantly improved at adolescence.

## Discussion

In the present study, we assessed the effect of dual therapy hypothermia + allopurinol (HIHA) after moderate focal HI in P10 rats. We found signs of reduced apoptosis and a global improvement in the neuropathological score and functional outcome in all treatment groups (HIA, HIH, and HIHA). Moreover, a sex-specific benefit of allopurinol treatment was detected. Females from the allopurinol-treated groups showed decreased markers of apoptosis and better water maze performance than males with the same treatment (HIA, HIHA).

TH intervenes in many pathways after HI injury, reducing metabolic rate and glutamate release, decreasing oxygen species, and regulating expression of inflammatory and apoptic cascades [35]. Despite the implementation of TH as a standard of care in many clinical units for HIE [12], it does not provide complete neuroprotection [36], making further investigation into additional therapies to improve neurological outcomes necessary. Xenon [16], Epo [37,38], N-Acetylcysteine (NAC) [34], and cannabidiol [39] are some of the drugs that have been tested in experimental models to potentiate the effects of TH. Historically, one of the first strategies evaluated for HIE treatment was allopurinol, an antioxidant molecule reported to act as a mild neuroprotective agent [40].

Oxidative stress is involved in HI injury physiopathology [41]. The neonatal brain handles oxidative stress poorly, with antioxidant activity less than half of adult levels [42,43]. One of the most important pro-oxidant pathways of free radical production is xanthine oxidase (XO) [44]. XO enzyme plays a key role in the high-energy phosphate system [45]. In physiological conditions, it coexists with xanthine dehydrogenase (XDH). Many investigators agree that XDH activity is converted by sulfhydryl oxidation or limited proteolysis to an oxidase that produces superoxide and hydrogen peroxide. It is worth noting, nevertheless, that both XO and XDH can oxidize NADH, with concomitant formation of reactive oxygen species [46,47]. Physiologically, XO and XDH participate in a variety of biochemical reactions including the hydroxylation of various purines, pterins, and aromatic heterocycles, thereby contributing to the detoxification or activation of endogenous compounds and xenobiotics. One of XOR’s primary roles is the conversion of hypoxanthine to xanthine and xanthine to uric acid. Allopurinol, a xanthine oxidase inhibitor, acts by interrupting the conversion of hypoxanthine into xanthine and uric acid, limiting the production of toxic reactive oxygen species [19,27]. In addition, allopurinol is a chelator of non-bound protein iron (NBPI) and a direct scavenger of hydroxyl free radicals [48]. All these properties suggest it should be useful as a neuroprotective agent. Palmer and collegues demonstrated its neuroprotective effects in P7 rats after HI [19]. When it was tested in humans, short-term outcome improvement could not be confirmed [49]. Apparently, no advantage from neonatal treatment was seen when the interval of treatment initiation was very long. Despite this, some years later, when long-term outcome was evaluated (at 4–8 years) in those babies [49], moderately asphyxiated infants treated with allopurinol presented a reduction in the risk of death or severe disability [25].

As has been pointed out in many studies, the major drawback to postasphyxial hypothermia or pharmacological treatment is the small therapeutic window in which treatment must be initiated. This is even truer if the aim is to avoid or reduce free radical stress. The optimal point in time to start antioxidative treatment is at birth or even before birth. Trying to simulate this condition, many authors have suggested that antenatal administration, during labor, could amplify its effects. Torrance and colleagues reported a decrease in p-100B in plasma leves in those fetuses that had received antenatal allopurinol when an HI situation was suspected [50]. Some years later, a randomised controlled trial suggested that allopurinol had a potential neuroprotective effect in girls as indicated by lower S100β and neuroketal values in the treatment group [51].

Our study reveals that the combination of hypothermia + allopurinol after moderate focal HI in P10 rats decreases infarct and improves residual brain volumes at 72 h, although in males it does not provide more neuroprotection than hypothermia alone. Infarct volume at 72 h was reduced from 33.60% in the untreated group to 13.41% in HIA, 5.1% in HIH, and 3.8% in HIHA. These results are in accordance with the first reports on hypothermia in which the infarct area was reduced to about 50% when compared with the vehicle [52]. In those initial papers, no sex differences were reported.

In the same line, in our study, the hippocampal volume was better preserved in HIA, HIH, and HIHA females than in HI. Interestingly, allopurinol administration seemed to increase the neuroprotective effect in females, not only in those that received combined therapy (HIHA), but also in those females that received it without TH (HIA) when compared to HI. It is important to point out that in our study allopurnol administration was very early, less than 15 minutes after the HI injury, in order to avoid radical production, thereby simulating the situation that seems to happen when it is administered during labor ([51]. These results are also in accordance with what Nie and colleages reported with another antioxidant therapy, N-acetylcysteine (NAC), suggesting that antioxidants may provide less neuroprotection in males [38]. Sex differences have been reported in neonatal models of hypoxic-ischaemic brain injury [53]. Additionally, in humans, male infants are more vulnerable to perinatal insult, suffering worse long-term cognitive deficits compared to females with equivalent injury [54,55,56]. Recent studies have demonstrated that mechanisms of cell death vary depending on sex [57,58].

After an HI event, morphological features of three different cell death types can be observed: necrotic, apoptotic, and autophagic cell death [59,60]. The best known cell death forms are necrosis and apoptosis, even though the importance of auhophagy-mediated cell death has recently become a field of interest [61,62]. Apoptosis has been well described as a programmed delayed cell death mechanism with different intracellular signaling pathways. Complete understanding of this process represents a potential target for neuroprotective interventions.

Although there are multiple pathways to programmed cell death, caspase-3 is the final ‘executioner,’ and thus caspase-3 activation may be used as a reasonable marker of apoptosis. It has been demonstrated that hypothermia reduces caspase-3 expression in newborn rats [63]. In our animals, caspase-3 was increased in HI animals 24h post-injury when compared to the other groups, as has previously been demonstrated [64]. Supporting histological results, there was also a decrease in caspase-3 activity in females that received allopurinol (HIHA, HIA). Oxidant reagents can lead to the activation of caspases, triggering apoptosis [65]. Our results suggest that allopurinol administration alone or in combination with hypothermia may reduce free radical liberation, decreasing caspase activation and consequently reducing brain injury. This process, as explained above, seems to be more pronounced in females. This is in accordance with a recently published hypothesis suggesting that programmed cell death in females depends mainly on caspase activation [30], as well as on their greater sensitivity to redox status.

Regarding functional performance, we found the same tendency in histological, molecular, and learning and memory tests. Treated animals (HIA, HIH, HIHA) had better results in WMT. When this was analysed taking gender into account, female allopurinol-treated (alone or in combination with hypothermia) animals achieved better results than males, again. The functional recovery was almost complete.

Despite these data, we found no consistent results to support the idea that hypothermia + allopurinol was better than hypothermia alone. Nevertheless, it is important to point out that the effect of both treatments independently was very significant, and therefore it is quite difficult to gain even more neuroprotection, especially with so few animals.

Dual therapy has a good neuroprotection spectrum. Other outcomes and molecular issues should be evaluated to test their potential benefits when compared to hypothermia alone.

## Conclusions

There is increasing evidence that neuroprotective strategies may have different effects depending on sex [29]. Our results also support this notion and, together with other published data, invite the reasonable conclusion that gender plays an important role in the effectiveness of each therapy. Those therapies that act by inhibiting the caspase-dependent pathway would have better results in females, and even more so if they were antioxidant therapies. Therefore, it seems reasonable that to achieve better neuroprotection, several therapeutic strategies should be used, and many individual considerations such as gender should be taken into account to indicate the best therapy for each patient.

## Supporting information

S1 Fig Negative geotaxis test results. All treated groups presented better results than the HI ones. No differences in sex were detected.
(TIF)
