# Supplementary figures and images for: Neuroprotection with hypothermia and allopurinol in an animal model of hypoxic-ischemic injury: Is it a gender question?
Source: PLoS One. 2017 Sep 20;12(9):e0184643. doi: 10.1371/journal.pone.0184643 (PMC5606927; doi:10.1371/journal.pone.0184643)

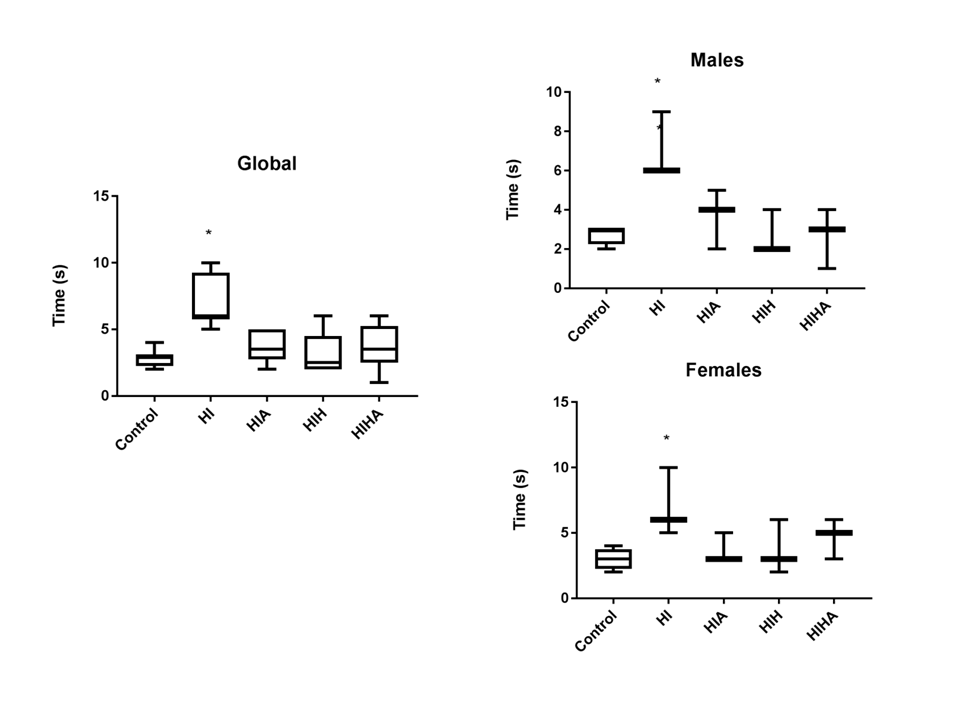

Supplement: S1 Fig — All treated groups presented better results than the HI ones. No differences in sex were detected. (TIF) [file pone.0184643.s001.tif]
